# Identification of Rumen Microbial Genes Involved in Pathways Linked to Appetite, Growth, and Feed Conversion Efficiency in Cattle

**DOI:** 10.3389/fgene.2019.00701

**Published:** 2019-08-08

**Authors:** Joana Lima, Marc D. Auffret, Robert D. Stewart, Richard J. Dewhurst, Carol-Anne Duthie, Timothy J. Snelling, Alan W. Walker, Tom C. Freeman, Mick Watson, Rainer Roehe

**Affiliations:** ^1^Beef and Sheep Research Centre, Future Farming Systems Group, Scotland’s Rural College, Edinburgh, United Kingdom; ^2^Division of Genetics and Genomics, The Roslin Institute and R(D)SVS, University of Edinburgh, Edinburgh, United Kingdom; ^3^The Rowett Institute, University of Aberdeen, Aberdeen, United Kingdom

**Keywords:** feed conversion efficiency, appetite, metagenomics, rumen microbiome, microbial gene networks

## Abstract

The rumen microbiome is essential for the biological processes involved in the conversion of feed into nutrients that can be utilized by the host animal. In the present research, the influence of the rumen microbiome on feed conversion efficiency, growth rate, and appetite of beef cattle was investigated using metagenomic data. Our aim was to explore the associations between microbial genes and functional pathways, to shed light on the influence of bacterial enzyme expression on host phenotypes. Two groups of cattle were selected on the basis of their high and low feed conversion ratio. Microbial DNA was extracted from rumen samples, and the relative abundances of microbial genes were determined *via* shotgun metagenomic sequencing. Using partial least squares analyses, we identified sets of 20, 14, 17, and 18 microbial genes whose relative abundances explained 63, 65, 66, and 73% of the variation of feed conversion efficiency, average daily weight gain, residual feed intake, and daily feed intake, respectively. The microbial genes associated with each of these traits were mostly different, but highly correlated traits such as feed conversion ratio and growth rate showed some overlapping genes. Consistent with this result, distinct clusters of a coabundance network were enriched with microbial genes identified to be related with feed conversion ratio and growth rate or daily feed intake and residual feed intake. Microbial genes encoding for proteins related to cell wall biosynthesis, hemicellulose, and cellulose degradation and host–microbiome crosstalk (e.g., *aguA, ptb*, K01188, and *murD*) were associated with feed conversion ratio and/or average daily gain. Genes related to vitamin B12 biosynthesis, environmental information processing, and bacterial mobility (e.g., *cobD*, *tolC*, and *fliN*) were associated with residual feed intake and/or daily feed intake. This research highlights the association of the microbiome with feed conversion processes, influencing growth rate and appetite, and it emphasizes the opportunity to use relative abundances of microbial genes in the prediction of these performance traits, with potential implementation in animal breeding programs and dietary interventions.

## Introduction

The global population is expected to reach 9.8 billion by 2050 (United Nations–Department of Economic and Social Affairs/Population Division, 2017), resulting in an escalation of the global demand for food and of the need for economically and environmentally sustainable livestock production systems (Godfray et al., 2010; Gerber et al., 2013). A large portion of livestock production is based on ruminants. In 2017, the EU-28 had a population of 88 million bovine animals, including cattle and water buffalo (Eurostat, 2018). Ruminants are particularly interesting due to their ability to convert human-indigestible plant biomass into high-quality products for human consumption such as meat and milk. Ruminants live in a symbiotic relationship with their rumen microbiota (comprising bacteria, protozoa, fungi, and archaea), which produce enzymes able to digest their food by breaking down complex polysaccharides of the plant biomass into volatile fatty acids (VFA), microbial proteins, and vitamins (Russell and Hespell, 1981; Bergman, 1990; Van Soest, 1994). Thus, the rumen microbiota fermentation profile has a significant influence on the feed conversion efficiency of the host (Russell, 2001; Li et al., 2009; Hernandez-Sanabria et al., 2011; Jami et al., 2014; Sasson et al., 2017; Meale et al., 2018) and is accountable for up to 70% of the host’s daily energy requirements (Bergman, 1990).

In beef cattle production systems, expenses associated with feed account for up to 75% of the total production costs (Moran, 2005a; Nielsen et al., 2013), which makes the improvement of feed conversion efficiency very economically compelling. There is consequently great interest in understanding the host–microbial symbiotic relationships responsible for the conversion of feed into energy, protein, and vitamins usable by the host animal, but the mechanisms and degree to which the rumen microbiome impacts on animal production, health, and efficiency remain undercharacterized (Brulc et al., 2009; Creevey et al., 2014). Although the rumen harbors a core microbiome (Jami and Mizrahi, 2012; Henderson et al., 2015), in agreement with studies performed in the human gastrointestinal tract (Tap et al., 2009; Qin et al., 2010), the structure, and composition of the rumen microbiome varies within and between animals with differing performance traits. For example, in lactating dairy cattle, the increased methane yield during late lactation in comparison to early lactation within the same individual was found to be associated with significant changes in the ruminal microbial community structure (Lyons et al., 2018); Myer et al. (2015) showed different relative abundances of some microbial taxa and operational taxonomic units in animals with different average daily gain (ADG); Shabat et al. (2016) focused on residual feed intake (RFI) to demonstrate that highly efficient animals had a less diverse microbiota, being dominated by specific taxa and microbial genes which were involved in simpler metabolic pathway networks when compared to their less efficient counterparts. Other authors have reported that the rumen microbiome varies more between animals than within animals, proposing that the host itself and its physiological parameters have a significant influence on its own rumen microbiome (Li et al., 2009) and, therefore, on the efficiency of feed conversion into energy. In a mouse study, Benson et al. (2010) found that there is a well-defined portion of the gut microbiota that is subject to host genetic control, proposing it to be regarded as a host trait, rather than an environmental trait affecting the host. In agreement, in a beef cattle study, Roehe et al. (2016) confirmed the host genetic influence on the rumen bacterial composition using a genetic model based on sire progeny groups. The differences between sire progeny groups in methane emissions were in some cases larger than the differences found between diets differing largely in plant fiber content, suggesting a substantial host genetic influence on the microbial communities.

Selecting animals for breeding based on their ability to harvest energy from feed, together with nutritional interventions, could be the basis for an effective strategy to produce faster growing and more efficient animals (Gerber et al., 2013; Scollan et al., 2018). Given that the host has influence over the ruminal microbiome, which impacts the animals’ feed conversion efficiency, this selection may be further improved by the inclusion of rumen metagenomic information into predictive models, as previously suggested by Ross et al. (2013). Feed conversion efficiency is very often estimated by either feed conversion ratio (FCR) or RFI; the latter is independent of growth and maturity patterns and is expected to be more sensitive and precise in measurements of feed utilization (Arthur and Herd, 2008). The use of microbial genes as proxies for feed conversion efficiency traits may be much more cost effective, rapid, and less labor intensive than their recording (Ross et al., 2013; Roehe et al., 2016). Our earlier research was the first proposing that the inclusion of relative abundance of microbial genes as proxies for FCR may be favorable, allowing their use as selection criteria for breeding animals, by identifying 49 microbial genes that explained 88.3% of the variation observed in FCR (Roehe et al., 2016). To our knowledge, no other studies have focused on the relationship between microbial gene abundances and RFI, daily feed intake (DFI), and ADG, which highlights the importance and novelty of the present work.

This study aimed at validating whether rumen microbial gene abundances are suitable proxies for feed conversion efficiency traits such as FCR; the analysis was further extended by focusing on RFI. Based on the previous evidence of strong interactions between the rumen microbiome and the host animal with consequences for feed conversion efficiency (Guan et al., 2008; Roehe et al., 2016; Shabat et al., 2016), we hypothesized that microbial gene abundances are linked to the animals’ appetite and, consequently, to feed intake. A further aim of this research was to gain insight into the association of growth rate with the microbial gene abundances. Building on this, we aimed at better understanding the rumen microbial functional network associated with feed conversion efficiency and its component traits. This research will improve on the current knowledge about the impact of the rumen microbiome on appetite, growth, and efficiency of feed conversion processes.

## Materials and Methods

### Ethics Statement

This study was conducted at the Beef and Sheep Research Centre, SRUC, UK. The study was carried out in accordance with the requirements of the UK Animals (Scientific Procedures) Act 1986. The protocol was approved by the Animal Experiment Committee of SRUC. All standard biosecurity and institutional safety procedures were applied during the animal experiment and the laboratory analysis.

### Animals, Adaptation Period, and Measurement of Traits

Two experiments were carried out to determine the effect of nitrate or lipid additives within different basal diets on methane emissions from beef cattle. The first experiment was conducted in 2013, and it consisted of a 2 × 2 × 3 factorial design including 84 steers of two breed types (crossbreed Charolais, CHx and Luing); two basal diets, forage (FOR) and concentrate (CONC), which consisted respectively of ratios of 520:480 and 84:916 forage to concentrate (g/kg dry matter); and three treatments, nitrate and lipid feed additives, as well as the control. From these animals, 24 animals were selected with extreme high and low FCR values within breed type and basal diet (two animals per feed additive and control). More details related to this experiment can be found in Duthie et al. (2015) and Troy et al. (2015). The second experiment was a 2 × 4 factorial design experiment, conducted in 2014, involving 80 animals. There were two breed types—40 crossbred Limousin (LIMx) and 40 crossbred Aberdeen Angus (AAx)—which were subject to a balanced design consisting of four dietary treatments using one basal diet (550:450 forage to concentrate ratio g/kg dry matter, FOR) and testing the effects of feed additives nitrate, lipid, or their combination in comparison to the control on methane output. Full details of the experiment are presented in Duthie et al. (2017). From this experiment, 18 animals were selected within each combination of breed type and diet: nine for the high FCR group and nine for the low FCR group. DFI was assessed by measuring dry matter intake (DMI, kg/day), which was recorded in both experiments using electronic feeding equipment (HOKO, Insentec, Marknesse, The Netherlands). Body weight (BW) was measured weekly using a calibrated weight scale (before fresh feed was offered). Growth was modeled by linear regression of BW against test date to obtain ADG, mid-test BW, and mid-test metabolic BW (MBW = BW^0.75^). FCR was calculated as average DMI (kg/day) divided by ADG. RFI was estimated as deviation of actual DMI (kg/day) from DMI predicted based on linear regression of actual DMI on ADG, mid-MBW, and fat depth at 12^th^/13^th^ rib at the end of the 56-day test (Duthie et al., 2015; Troy et al., 2015; Duthie et al., 2017).

**Figure 1:**
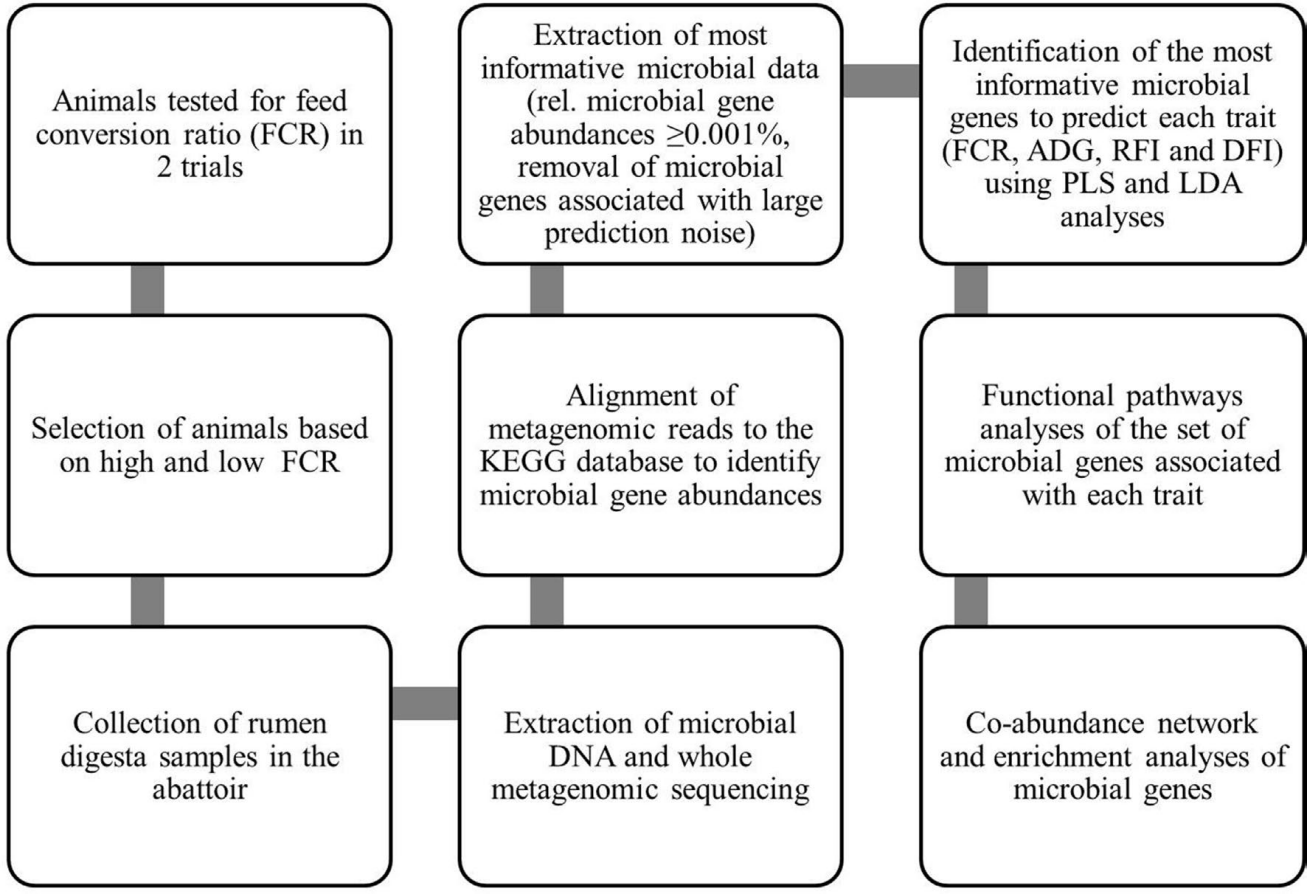
Flowchart summarizing methods for generation of data and their statistical analyses: This flowchart summarizes how the data were generated and which statistical analyses were used to identify the associations between gene abundances and performance traits of animals to understand the rumen microbial functional pathways associated with these traits. KEGG, Kyoto Encyclopedia of Genes and Genomes; FCR, feed conversion ratio; ADG, average daily gain; RFI, residual feed intake; DFI, daily feed intake; PLS, partial least squares; LDA, linear discriminant analysis.

A flowchart summarizing the methods for generation of data and subsequent statistical analyses is presented in **Figure 1**.

### Sampling of Rumen Digesta and Whole Metagenomic Sequencing

As described in Duthie et al. (2015) and Auffret et al. (2017), animals from both experiments were slaughtered in a commercial abattoir where two samples of rumen digesta (∼50 ml) were collected immediately after the rumen was opened to be drained. The slaughter house sample collection process results in well-mixed samples of rumen contents. DNA was extracted from the rumen samples of 42 animals following the methodology described in Rooke et al. (2014). Illumina TruSeq libraries were prepared from genomic DNA and sequenced on Illumina HiSeq systems 4000 by Edinburgh Genomics (Edinburgh, UK). Paired-end reads (2 × 150 bp) were generated, resulting in between 8 and 15 GB per sample (between 40 and 73 million paired reads). The raw data can be downloaded from the European Nucleotide Archive under accession PRJEB21624.

### Identification of the Rumen Microbial Gene Abundances

Bioinformatics analysis for identification of rumen microbial genes was carried out as previously described by Wallace et al. (2015). Briefly, to measure the abundance of known functional microbial genes in the rumen samples, reads from whole metagenome sequencing were aligned to the Kyoto Encyclopedia of Genes and Genomes (KEGG) database (Kanehisa and Goto, 2000) using Novoalign (www.novocraft.com). Parameters were adjusted such that all hits were reported that were equal in quality to the best hit for each read and allowing up to a 10% mismatch across the fragment. The KEGG Orthologue groups (KO) of all hits that were equal to the best hit were examined. If we were unable to resolve the read to a single KO, the read was ignored; otherwise, the read was assigned to the unique KO. Read counts were summed and normalized to the total number of hits. This mapping of the whole metagenomic data to the KEGG database resulted in a dataset comprising of 4,966 KEGG genes. Microbial genes were removed from the dataset when they were absent from three or more animals and when the mean relative abundance was lower than 0.001%, leaving 1,692 microbial genes for further analyses.

### Statistical Analysis

For each of the 1,692 microbial genes, a linear model was fitted, including as fixed effects a combined class variable of breed, diet, and year of experiment (six levels) and the FCR groups (high FCR, FCR-H and low FCR, FCR-L) using the lm() function in R version 3.4.2. The microbial genes which resulted in *P* ≥ 0.1 for the differences in FCR groups were not considered in the partial least squares analyses (PLS, SAS version 9.3 for Windows, SAS Institute Inc., Cary, NC, USA) to avoid excessive noise of microbial genes uncorrelated to the traits of interest. In the linear model, FCR groups were replaced successively by ADG, RFI, and DFI as covariables to identify only potentially relevant microbial genes of these traits for further PLS analyses. In addition, genes with unknown function were removed from these datasets.

Microbial genes whose relative abundances were significantly associated to each trait in the linear models were analyzed using a sequential PLS-based methodology. First, PLS models were calculated in which the number of latent variables was determined by “leave-one-out” cross-validation, and genes with lower variable importance in projection (VIP) were removed. Second, the sets of genes created in the first step were evaluated by PLS models using three latent variables to determine the smaller set of genes leading to higher explained variation of both independent and dependent variables.

Each set of microbial genes identified in the PLS analyses as best predicting the trait was then used in a linear discriminant analysis (LDA), performed in R version 3.5.1 (2018-07-02) package MASS_7.3-51.4. In these analyses, the categories were for FCR those described previously as FCR-H and FCR-L; for all other traits, animals were classified as high or low, depending on their observations being higher or lower than the median (balanced for trial, breed, and diet).

The microbial genes identified to be significantly associated with each trait were submitted to an extensive review about their functionality based on databases such as KEGG (Kanehisa Laboratories, 2018), BioCyc (Karp et al., 2017), and UniProt (Bateman, 2019) and information from the literature.

#### Networks

The coabundances between microbial genes were investigated in a stepwise network analysis using the Graphia Professional software (Kajeka Ltd, Edinburgh; Freeman et al., 2007), in which nodes represent microbial genes and edges represent a correlation value above a defined value of *r*. In the first step, the correlation threshold of *r* = 0.45 was selected such that all microbial genes (*n* = 1,692) were included in the network. The microbial genes identified by PLS to be associated with a trait of interest were then located in the network. Clustering was performed using the Markov clustering method (MCL) available in Graphia Professional using the default settings (inflation, preinflation, and scheme values of 6). All clusters that held at least one microbial gene previously identified in the PLS analysis to be associated with a trait of interest were identified. These were incorporated into a new network generated at correlation threshold of *r* = 0.80 containing 1,135 microbial genes. MCL was then performed on this network, with inflation and preinflation values of 2 and scheme value of 6, reflecting the clustering structure suggested in the network itself. Analyses of enrichment of genes identified in the PLS as associated to each trait were performed on the clusters, and significance was assessed at *P* < 0.05.

## Results

### Performance Traits Related With Feed Conversion Efficiency

**Figure 2:**
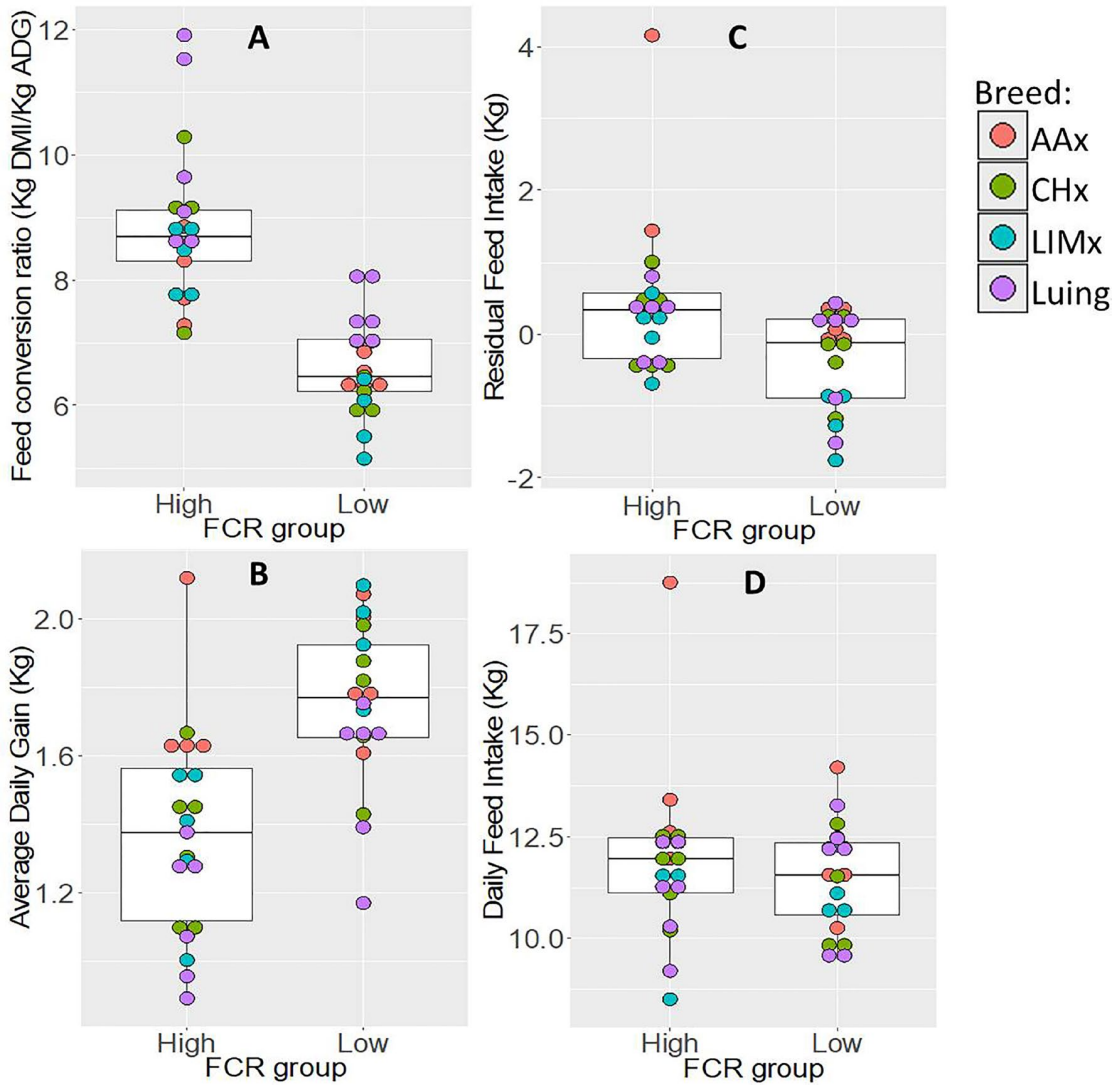
Distribution of variation and range of performance traits: **(A)** feed conversion ratio, **(B)** average daily gain, **(C)** residual feed intake, and **(D)** daily feed intake within feed conversion ratio groups (high and low). The boxplots show the variation and range of each trait within each feed conversion ratio group. FCR, feed conversion ratio; AAx, crossbred Aberdeen Angus; CHx, crossbred Charolais; LIMx, crossbred Limousin.

The average FCR values observed for animals selected into FCR-H (inefficient) and FCR-L (efficient) groups differed significantly by 2.3 kg DFI/kg ADG (**Figure 2**). When comparing these two groups for other traits, the FCR-H group had significantly higher values of RFI (0.8 kg) and significantly lower ADG (0.39 kg); in the case of DFI, no significant difference was observed between the FCR groups.

ADG and FCR had a strong significant negative correlation of 0.80, suggesting that high growth rate is associated with efficient animals, using less feed per kilogram of weight gain. FCR and RFI were significantly positively correlated, but at a low level of 0.32. DFI was significantly correlated with RFI and ADG at high and moderate levels of 0.77 and 0.53, respectively.

### Rumen Microbial Genes Associated With Feed Conversion Efficiency Traits

**Figure 3:**
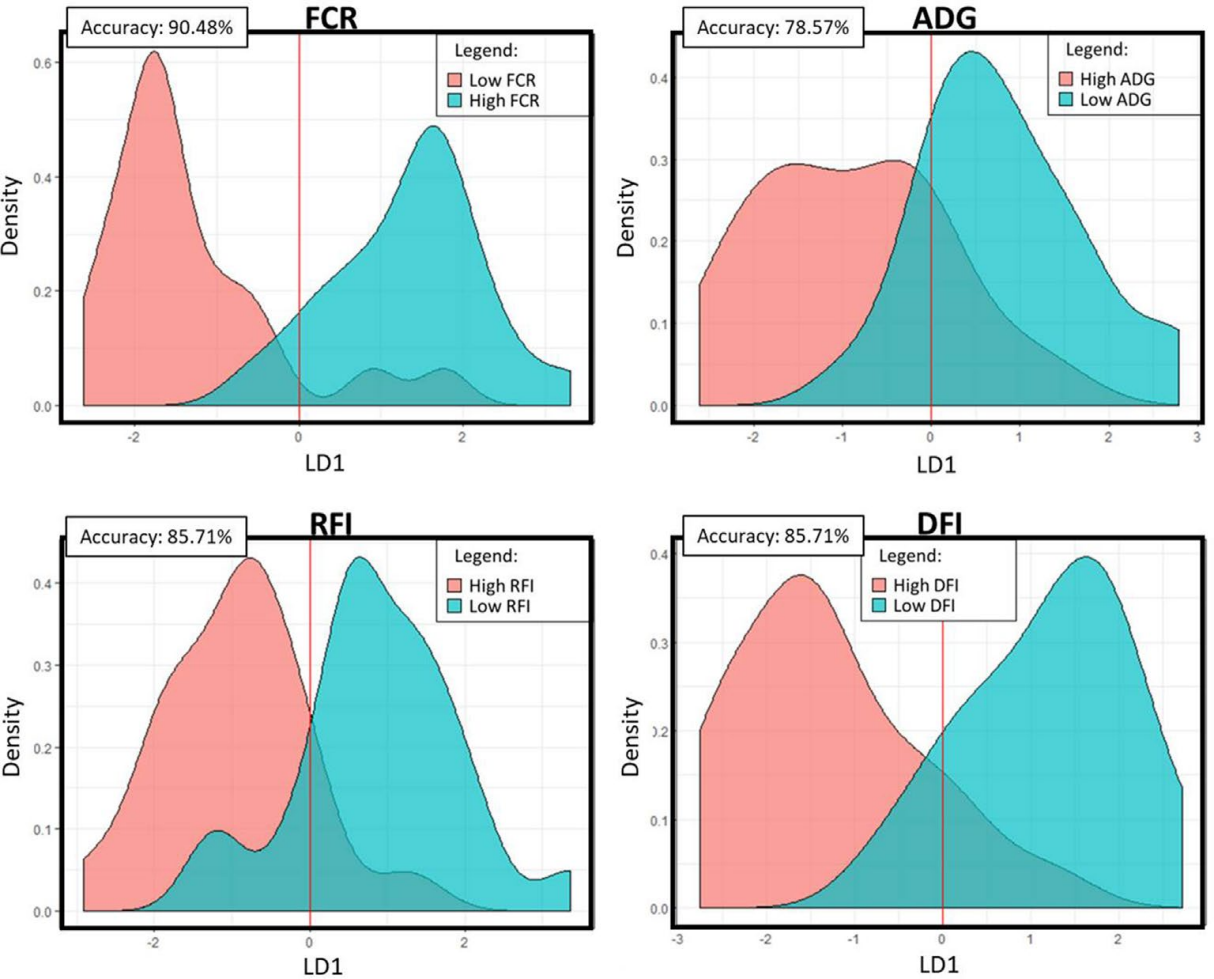
Linear discriminant analysis density plots: Microbial genes identified in the PLS analyses to be significantly associated with the trait were used in a linear discriminant analysis of high- and low-performing animals. The density plots represent the predicted categories for each trait. The accuracy value represents the percentage of animals that were correctly assigned to their category. FCR, feed conversion ratio; ADG, average daily gain; RFI, residual feed intake; DFI, daily feed intake; LD1, linear discriminant 1.

The PLS analyses identified sets of 20 and 14 microbial genes whose relative abundances explained 63.4 and 65.4% of the variation in FCR and ADG, respectively, and sets of 17 and 18 microbial genes whose relative abundances explained 65.6 and 72.9% of the variation in RFI and DFI, respectively, including the combined fixed effect of diet, breed, and year of experiment (**Table 1**). Without this combined fixed effect, the variances explained by microbial genes in FCR and ADG decreased to 54.2 and 61.4%, while in RFI and DFI, they decreased to 50.8 and 67.7%, respectively. A discriminant analysis between groups of high- and low-performing animals, using the set of microbial genes identified in the PLS analysis to best predict each trait, resulted in prediction accuracies of 90, 79, 86, and 86% for FCR, ADG, RFI, and DFI (**Figure 3**).

**Table 1:** Percentage of variation in each trait explained by the microbial genes identified in the partial least squares (PLS).

		Percent variation accounted for by partial least squares factors			
		Model effects		Dependent variables	
Trait	No. factors	Current	Total	Current	Total
FCR	1	41.59	41.59	35.46	35.46
	2	6.35	47.94	21.19	56.65
	3	7.57	55.51	6.72	63.37
ADG	1	39.42	39.42	49.26	49.26
	2	9.60	49.02	11.47	60.73
	3	7.97	56.99	4.67	65.40
RFI	1	24.04	24.04	44.32	44.32
	2	13.95	37.99	16.80	61.12
	3	16.72	54.71	4.52	65.63
DFI	1	28.98	28.98	44.94	44.94
	2	21.25	50.23	19.94	64.88
	3	7.86	58.09	8.05	72.93

The number of factors refers to the number of latent variables in which the total number of microbial genes (independent variables) were projected in the PLS procedure, and each factor accounts for a portion of the total explained variation. The “Model Effects” columns refer to the percent variability of the independent variables matrix that relates to the respective percent variability presented in the “Dependent Variables” columns. The “Current” columns present values for each extracted factor individually, and the “Total” columns present the subtotal variation. The cells colored in gray contain the values of percent variation explained by the three latent variables for each trait. FCR, feed conversion ratio; ADG, average daily gain; RFI, residual feed intake; DFI, daily feed intake.

**Figure 4:**
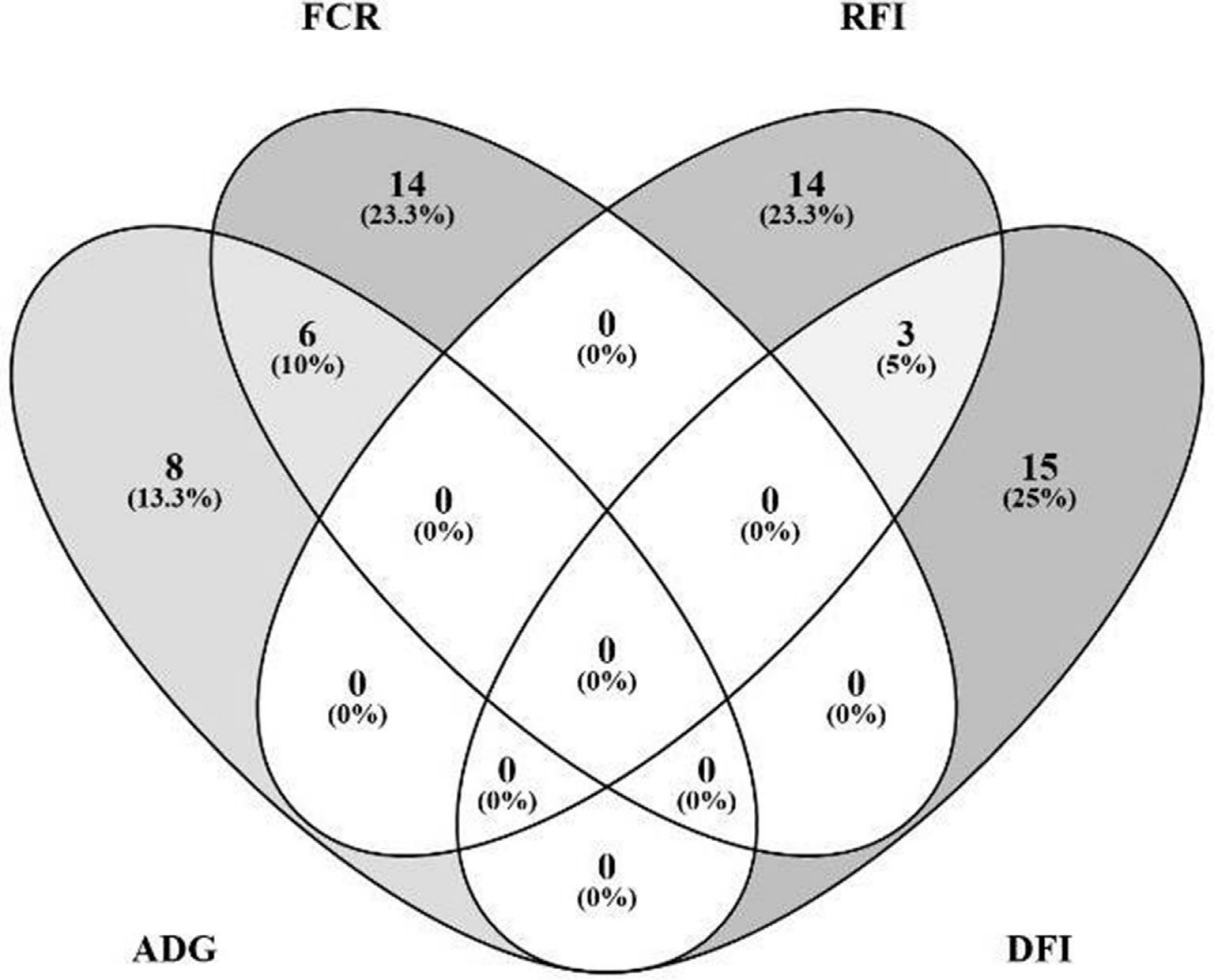
Overlap analysis of identified microbial genes: The image illustrates the number of microbial genes identified in the partial least squares analysis as fitted for prediction of each animal performance trait exclusively, and the number of microbial genes simultaneously predicted for multiple traits: six microbial genes were simultaneously identified for prediction of FCR (feed conversion ratio) and ADG (average daily gain), and three for both RFI (residual feed intake) and DFI (daily feed intake).

The Venn diagram presented in **Figure 4** illustrates the overlap between the sets of genes identified for the prediction of each of the four traits. For the prediction of FCR and ADG, six microbial genes were simultaneously selected: UDP-N-acetylmuramoylalanine-D-glutamate ligase, glycine cleavage system H protein, translation initiation factor IF-1, N utilization substance protein A, DNA-binding protein HU-beta, and diphthamide synthase subunit *dph2* (*murD*, *gcvH*, *infA*, *nusA*, *hupB*, and *dph2*, respectively). Three microbial genes were simultaneously selected for the prediction of traits RFI and DFI: glucose-1-phosphate cytidylyltransferase, CDP-glucose 4,6-dehydratase, and energy-converting hydrogenase B subunit D (*rfbF*, *rfbG*, and *ehbD*, respectively). The microbial genes identified for the prediction of more than one trait are highlighted in the shaded rows in **Tables 2**–**5**, in which a more detailed information about their function and importance for prediction is provided.

**Table 2:** Summary of microbial genes identified for the prediction of FCR.

KEGG id	Description	Gene name abbreviation	Pathways	Mean abundance	PLS estimate	VIP	Cluster
K03783	Purine-nucleoside phosphorylase	*punA*	^2^Metabolic pathways; biosynthesis of secondary metabolites; purine metabolism; pyrimidine metabolism; nicotinate and nicotinamide metabolism	0.0107	−0.2755	1.22	^1^
K08138	MFS transporter, SP family, xylose:H+ symporter	*xylE*	^3^Carbohydrate transport and metabolism, amino acid transport and metabolism, Inorganic ion transport and metabolism	0.0404	0.1135	1.09	05
K00046	Gluconate 5-dehydrogenase	*idnO*	^4^L-idonate degradation	0.0845	0.0166	1.08	11
K00040	Fructuronate reductase	*uxuB*	^2^Metabolic pathways; pentose and glucuronate interconversions	0.0847	0.0503	1.01	25
K01759	Lactoylglutathione lyase	*glo1*	^2^Pyruvate metabolism	0.0021	0.1547	1.01	09
K00849	Galactokinase	*galK*	^2^Metabolic pathways; galactose metabolism; amino sugar and nucleotide sugar metabolism	0.0631	0.0675	1.00	05
K01195	Beta-glucuronidase	*uidA*	^2^Metabolic pathways; biosynthesis of secondary metabolites; pentose and glucuronate interconversions; glycosaminoglycan degradation; porphyrin and chlorophyll metabolism; flavone and flavonol biosynthesis; drug metabolism—other enzymes; lysosome	0.0127	−0.1174	0.99	07
K14220	tRNA Asn	*tRNA-Asn*	^2^Aminoacyl-tRNA biosynthesis	0.0155	0.0139	0.96	NC
K00677	UDP-N-acetylglucosamine acyltransferase	*lpxA*	^2^Metabolic pathways; lipopolysaccharide biosynthesis; cationic antimicrobial peptide (CAMP) resistance	0.0403	−0.0186	0.91	25
K01188	Beta-glucosidase	beta-glucosidase	^2^Metabolic pathways; biosynthesis of secondary metabolites; cyanoamino acid metabolism; starch and sucrose metabolism; phenylpropanoid biosynthesis	0.0398	−0.0210	0.90	^1^
K07214	Enterochelin esterase and related enzymes	*fes*	^3^Inorganic ion transport and metabolism	0.0475	−0.1511	0.90	^1^
K03303	Lactate permease	*lctP*	^5^Lactate transmembrane transporter activity	0.0195	−0.0271	0.88	28
K00634	Phosphate butyryltransferase	*ptb*	^2^Metabolic pathways; butanoate metabolism	0.0075	−0.0079	0.85	^1^
K01235	Alpha-glucuronidase	*aguA*	^3^Carbohydrate transport and metabolism	0.0104	−0.0626	0.80	NC
K07561	diphthamide synthase subunit DPH2	*dph2*	^3^Translation, ribosomal structure and biogenesis	0.0030	−0.3881	1.86	01
K01925	UDP-N-acetylmuramoylalanine–D-glutamate ligase	*murD*	^2^Metabolic pathways; D-Glutamine and D-glutamate metabolism; peptidoglycan biosynthesis	0.0620	−0.0857	0.99	^1^
K02437	Glycine cleavage system H protein	*gcvH*	^2^Metabolic pathways; biosynthesis of secondary metabolites; biosynthesis of antibiotics; glycine, serine and threonine metabolism; glyoxylate and dicarboxylate metabolism; carbon metabolism	0.0069	−0.0167	0.93	^1^
K03530	DNA-binding protein HU-beta	*hupB*	^3^DNA binding protein: replication, recombination, and repair	0.0331	−0.0892	0.89	19
K02600	N utilization substance protein A	*nusA*	^3^Transcription	0.1126	−0.0655	0.89	^1^
K02518	Translation initiation factor IF-1	*infA*	^3^Translation, ribosomal structure and biogenesis	0.0346	0.0170	0.88	21

Each column respectively presents information about: 1) KEGG identifier, 2) description of the gene (from KEGG), 3) gene name abbreviation, 4) metabolic pathways in which this gene participates, 5) mean relative abundance of the microbial gene in 42 animals, 6) the partial least squares (PLS) estimate of the regression coefficient using three latent variables, 7) the variable importance in projection (VIP) calculated during the PLS analysis using three latent variables, and 8) the cluster in which the microbial gene was allocated in the final network. 1Microbial genes excluded from the final network due to the 0.80 minimum correlation threshold. NC, Microbial genes not clustered in the final network. Information retrieved from: 2KEGG database, 3NCBI database, 4BioCyc database, and 5UniProt database. The genes in this table explained 63.4% of the variation in FCR (feed conversion ratio). Rows colored in gray correspond to genes simultaneously identified for both FCR and ADG (average daily gain) prediction.

**Table 3:** Summary of microbial genes identified for the prediction of ADG.

KEGG id	Description	Gene name abbreviation	Pathways	Mean abundance	PLS estimate	VIP	Cluster
K01448	N-acetylmuramoyl-L-alanine amidase	*amiABC*	^2^Cationic antimicrobial peptide (CAMP) resistance	0.0236	−0.1937	1.22	06
K00133	Aspartate-semialdehyde dehydrogenase	*asd*	^2^Metabolic pathways; microbial metabolism in diverse environments; biosynthesis of secondary metabolites; biosynthesis of antibiotics; glycine, serine and threonine metabolism; monobactam biosynthesis; cysteine and methionine metabolism; lysine biosynthesis; 2-oxocarboxylic acid metabolism; biosynthesis of amino acids	0.1197	−0.0684	1.20	NC
K01912	Phenylacetate-CoA ligase	*paaK*	^2^Microbial metabolism in diverse environments; phenylalanine metabolism; biofilm formation—*Vibrio cholerae*	0.1543	−0.0980	1.16	16
K02919	Large subunit ribosomal protein L36	*rpmJ*	^2^Ribosome	0.0261	−0.1884	1.04	NC
K02879	Large subunit ribosomal protein L17	*rplQ*	^2^Ribosome	0.0773	0.0746	1.00	21
K02113	F-type H+-transporting ATPase subunit delta	*atpH*	^2^Metabolic pathways; oxidative phosphorylation; photosynthesis	0.0292	−0.0486	1.00	21
K00283	Glycine dehydrogenase subunit 2	*gcvPB*	^2^Metabolic pathways; biosynthesis of secondary metabolites; biosynthesis of antibiotics; glycine, serine and threonine metabolism; glyoxylate and dicarboxylate metabolism; carbon metabolism	0.0284	0.0502	0.99	25
K03775	FKBP-type peptidyl-prolyl cis-trans isomerase SlyD	*slyD*	^5^Posttranslational modification, protein turnover, chaperones	0.0139	0.0672	0.93	22
K07561	Diphthamide synthase subunit DPH2	*dph2*	^5^Translation, ribosomal structure, and biogenesis	0.0030	0.2310	1.20	01
K01925	UDP-N-acetylmuramoylalanine–D-glutamate ligase	*murD*	^2^Metabolic pathways; D-glutamine and D-glutamate metabolism; peptidoglycan biosynthesis	0.0620	0.1155	1.15	^1^
K02437	Glycine cleavage system H protein	*gcvH*	^2^Metabolic pathways; biosynthesis of secondary metabolites; biosynthesis of antibiotics; glycine, serine and threonine metabolism; glyoxylate and dicarboxylate metabolism; carbon metabolism	0.0069	0.1209	1.08	^1^
K03530	DNA-binding protein HU-beta	*hupB*	^5^DNA binding protein: replication, recombination, and repair	0.0331	0.1062	1.07	19
K02600	N utilization substance protein A	*nusA*	^5^Transcription	0.1126	0.0726	1.02	^1^
K02518	Translation initiation factor IF-1	*infA*	^5^Translation, ribosomal structure, and biogenesis	0.0346	0.0646	0.98	21

Each column respectively presents information about: 1) KEGG identifier, 2) description of the gene (from KEGG), 3) gene name abbreviation, 4) metabolic pathways in which this gene participates, 5) mean relative abundance of the microbial gene in 42 animals, 6) the partial least squares (PLS) estimate of the regression coefficient using three latent variables, 7) the variable importance in projection (VIP) calculated during the PLS analysis using three latent variables, and 8) the cluster in which the microbial gene was allocated in the final network. 1Microbial genes excluded from the final network due to the 0.80 minimum correlation threshold. NC, Microbial genes not clustered in the final network. Information retrieved from: 2KEGG database, 3NCBI database, 4BioCyc database, and 5UniProt database. The genes in this table explained 65.4% of the variation in ADG (average daily gain). Rows colored in gray correspond to genes simultaneously identified for both FCR (feed conversion ratio) and ADG prediction.

**Table 4:** Summary of microbial genes identified for the prediction of RFI.

KEGG id	Description	Gene name abbreviation	Pathways	Mean abundance	PLS estimate	VIP	Cluster
K03406	Methyl-accepting chemotaxis protein	*mcp*	^2^Two-component system; bacterial chemotaxis	0.0225	−0.0510	1.26	_1_
K03413	Two-component system, chemotaxis family, response regulator CheY	*cheY*	^2^Two-component system; bacterial chemotaxis	0.0018	0.0478	1.16	_1_
K01534	Cd2+/Zn2+-exporting ATPase	*zntA*	^5^Cation-transporting ATPase activity; metal ion binding; nucleotide binding	0.0211	−0.0653	1.16	04
K07258	serine-type D-Ala-D-Ala carboxypeptidase (penicillin-binding protein 5/6)	*dacC*	^2^Metabolic pathways; Peptidoglycan biosynthesis	0.0049	−0.0375	1.14	_1_
K07301	Cation:H+ antiporter	*yrbG*	^3^Inorganic ion transport and metabolism	0.0096	−0.0145	1.09	04
K04720	Threonine-phosphate decarboxylase	*cobD*	^2^Porphyrin and chlorophyll metabolism	0.0034	−0.0501	1.06	04
K03407	Two-component system, chemotaxis family, sensor kinase CheA	*cheA*	^2^Two-component system; bacterial chemotaxis	0.0048	−0.0236	1.04	_1_
K00595	Precorrin-6Y C5,15-methyltransferase (decarboxylating)	*cobL*	^2^Metabolic pathways; porphyrin and chlorophyll metabolism	0.0078	0.0223	1.02	04
K01571	Oxaloacetate decarboxylase, alpha subunit	*oadA*	^2^Metabolic pathways; pyruvate metabolism	0.0165	−0.0501	0.96	04
K02057	Simple sugar transport system permease protein	*ABC.SS.P*	^3^Carbohydrate transport and metabolism	0.0023	−0.1375	0.96	20
K02390	Flagellar hook protein FlgE	*flgE*	^2^Flagellar assembly	0.0015	−0.0376	0.87	_1_
K02417	Flagellar motor switch protein FliN/FliY	*fliN*	^2^Bacterial chemotaxis; flagellar assembly	0.0018	−0.1120	0.77	_1_
K03738	Aldehyde:ferredoxin oxidoreductase	*aor*	^2^Metabolic pathways; Microbial metabolism in diverse environments; Pentose phosphate pathway; Carbon metabolism	0.0144	−0.0657	0.68	NC
K02009	Cobalt transport protein	*cbiN*	^2^ABC transporters	0.0074	−0.1126	0.67	01
K01709	CDP-glucose 4,6-dehydratase	*rfbG*	^2^Metabolic pathways; amino sugar and nucleotide sugar metabolism	0.0041	0.2549	1.46	_1_
K00978	Glucose-1-phosphate cytidylyltransferase	*rfbF*	^2^Metabolic pathways; amino sugar and nucleotide sugar metabolism; starch and sucrose metabolism	0.0042	0.2056	1.23	_1_
K14113	Energy-converting hydrogenase B subunit D	*ehbD*	–	0.0010	0.1703	1.00	NC

Each column respectively presents information about: 1) KEGG identifier, 2) description of the gene (from KEGG), 3) gene name abbreviation, 4) metabolic pathways in which this gene participates, 5) mean relative abundance of the microbial gene in 42 animals, 6) the partial least squares (PLS) estimate of the regression coefficient using three latent variables, 7) the variable importance in projection (VIP) calculated during the PLS analysis using three latent variables, and 8) the cluster in which the microbial gene was allocated in the final network. 1Microbial genes excluded from the final network due to the 0.80 minimum correlation threshold. NC, Microbial genes not clustered in the final network. Information retrieved from: 2KEGG database, 3NCBI database, 4BioCyc database, and 5UniProt database. The genes in this table explained 65.6% of the variation in RFI (residual feed intake). Rows colored in grey correspond to genes simultaneously identified for both RFI and DFI (daily feed intake) prediction.

**Table 5:** Summary of microbial genes identified for the prediction of DFI.

KEGG id	Description	Gene name abbreviation	Pathways	Mean abundance	PLS estimate	VIP	Cluster
K00370	Nitrate reductase 1, alpha subunit	*narG*	^2^Microbial metabolism in diverse environments; nitrogen metabolism; two-component system	0.0022	−0.2272	1.22	^1^
K01858	Myo-inositol-1-phosphate synthase	*INO1*	^2^Metabolic pathways; biosynthesis of antibiotics; streptomycin biosynthesis; inositol phosphate metabolism	0.0542	−0.0459	1.14	^1^
K03685	Ribonuclease III	*rnc*	^2^Ribosome biogenesis in eukaryotes; proteoglycans in cancer	0.0288	−0.0097	1.13	^1^
K00613	Glycine amidinotransferase	*GATM*	^2^Metabolic pathways; glycine, serine and threonine metabolism; arginine and proline metabolism	0.0019	−0.1417	1.09	^1^
K02428	XTP/dITP diphosphohydrolase	*rdgB*	^2^Metabolic pathways; purine metabolism	0.0147	−0.0216	0.94	02
K03602	Exodeoxyribonuclease VII small subunit	*xseB*	^2^Mismatch repair	0.0035	0.0803	0.94	02
K03210	Preprotein translocase subunit YajC	*yajC*	^2^Bacterial secretion system; quorum sensing; protein export	0.0069	0.1317	0.93	^1^
K12340	Outer membrane channel protein TolC	*tolC*	^2^Beta-lactam resistance; cationic antimicrobial peptide (CAMP) resistance; two-component system; bacterial secretion system; plant−pathogen interaction; pertussis	0.0157	0.0068	0.92	02
K03043	DNA-directed RNA polymerase subunit beta	*rpoB*	^2^Metabolic pathways; purine metabolism; pyrimidine metabolism; RNA polymerase	1.2470	−0.0995	0.91	NC
K04751	Nitrogen regulatory protein P-II 1	*glnB*	^2^Two-component system	0.0151	0.0613	0.91	02
K03625	N utilization substance protein B	*nusB*	^3^Transcription termination	0.0135	0.0766	0.91	02
K06178	Ribosomal large subunit pseudouridine synthase B	*rluB*	^3^Translation, ribosomal structure, and biogenesis	0.0693	−0.0038	0.85	02
K05349	Beta-glucosidase	*bglX*	^2^Metabolic pathways; biosynthesis of secondary metabolites; cyanoamino acid metabolism; starch and sucrose metabolism; phenylpropanoid biosynthesis	0.2272	0.0063	0.84	^1^
K05515	Penicillin-binding protein 2	*mrdA*	^2^Peptidoglycan biosynthesis; beta-lactam resistance	0.0295	0.0214	0.82	02
K04764	Integration host factor subunit alpha	*ihfA*	^3^DNA binding: replication, recombination, and repair	0.0041	0.0306	0.80	02
K01709	CDP-glucose 4,6-dehydratase	*rfbG*	^2^Metabolic pathways; amino sugar and nucleotide sugar metabolism	0.0041	0.2412	1.53	^1^
K00978	Glucose-1-phosphate cytidylyltransferase	*rfbF*	^2^Metabolic pathways; amino sugar and nucleotide sugar metabolism; starch and sucrose metabolism	0.0042	0.2634	1.43	^1^
K14113	Energy-converting hydrogenase B subunit D	*ehbD*	–	0.0010	0.1594	1.16	NC

Each column respectively presents information about: 1) KEGG identifier, 2) description of the gene (from KEGG), 3) gene name abbreviation, 4) metabolic pathways in which this gene participates, 5) mean relative abundance of the microbial gene in 42 animals, 6) the partial least squares (PLS) estimate of the regression coefficient using three latent variables, 7) the variable importance in projection (VIP) calculated during the PLS analysis using three latent variables, and 8) the cluster in which the microbial gene was allocated in the final network. 1Microbial genes excluded from the final network due to the 0.80 minimum correlation threshold. NC, Microbial genes not clustered in the final network. Information retrieved from: 2KEGG database, 3NCBI database, 4BioCyc database, and 5UniProt database. The genes in this table explained 72.9% of the variation in DFI (daily feed intake). Rows colored in gray correspond to genes simultaneously identified for both RFI (residual feed intake) and DFI prediction.

**Figure 5:**
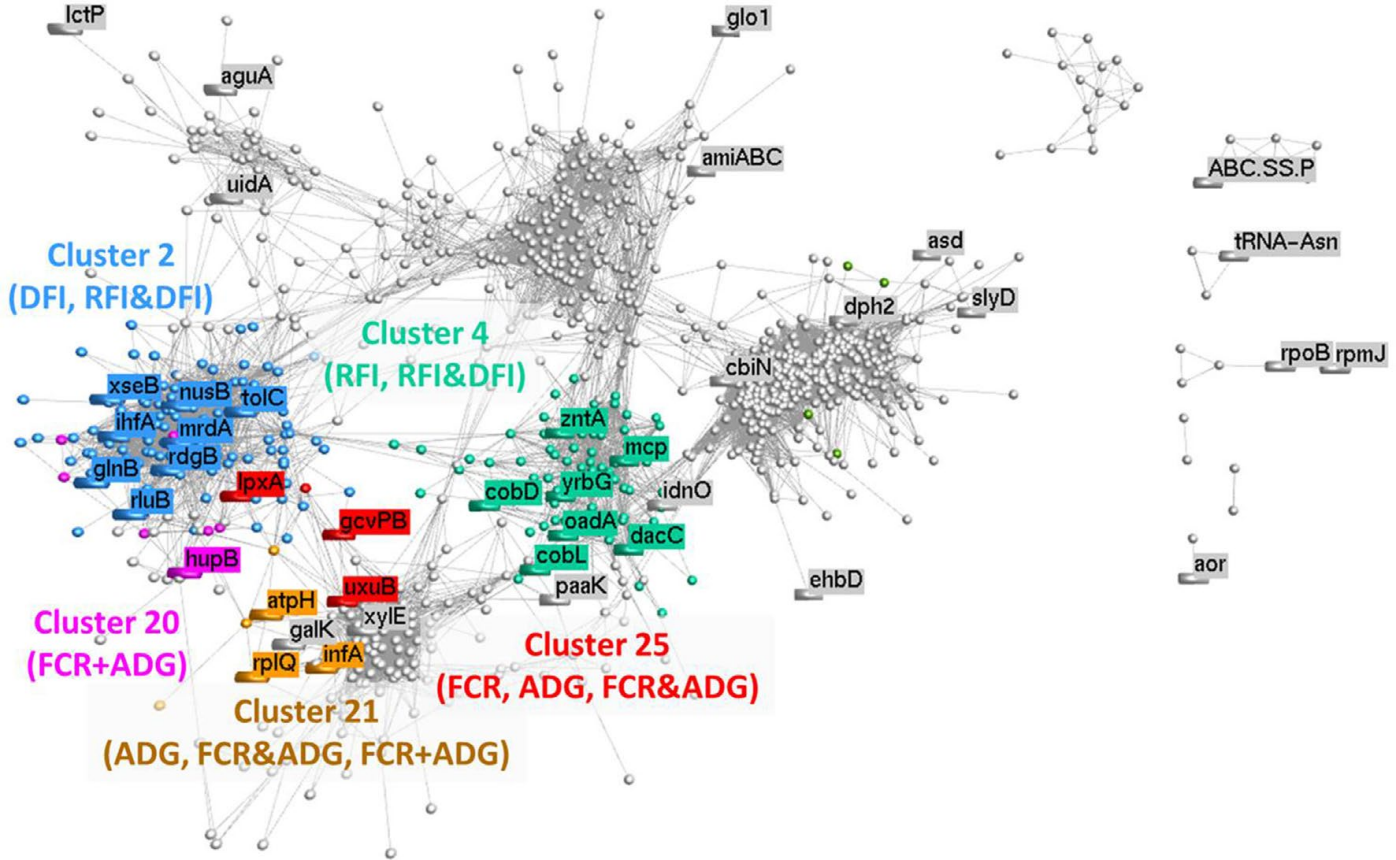
Correlation network analysis of metagenomic data: Each node represents a vector of relative abundances of each microbial gene in all 42 animals, and the edges represent a correlation between the microbial genes. A minimum correlation threshold of 0.80 was applied to the network. Different colors illustrate different clusters, which were calculated using MCL method (inflation: 2; preinflation: 2; scheme: 6). Clusters identified by numbers were found to be significantly (*P* < 0.05) enriched for microbial genes identified for the traits whose abbreviations are between brackets (FCR, feed conversion ratio; ADG, average daily gain; RFI, residual feed intake; DFI, daily feed intake; FCR&ADG, set including microbial genes identified for prediction of either FCR and/or ADG; RFI&DFI, set including microbial genes identified for prediction of RFI and/or DFI; FCR+ADG, set including microbial genes simultaneously identified for prediction of both traits FCR and ADG).

Based on the relative abundance of 1,135 microbial genes across rumen samples, a coabundance network was developed (**Figure 5**), and clusters were identified. The clustering pattern evidences the microbial genes that are more closely connected to microbial genes previously identified in the PLS analyses. The network cluster to which each microbial gene belongs to is presented in **Tables 2**–**5**. Cluster 2 was significantly enriched for microbial genes predicting DFI and RFI&DFI (RFI and/or DFI). Cluster 4 was enriched for microbial genes predicting RFI and RFI&DFI. Microbial genes simultaneously predicting FCR and ADG were enriched in clusters 20 and 21, while those predicting FCR&ADG (FCR and/or ADG) were enriched in clusters 21 and 25. ADG-predicting microbial genes were enriched in clusters 21 and 25, whereas FCR-predicting genes were only enriched in cluster 25. Other genes previously identified in the PLS analysis were scattered across the graph.

Most microbial genes identified exclusively for the prediction of FCR are related to carbohydrate metabolism and transport: fructuronate reductase, galactokinase, alpha-glucuronidase, beta-glucuronidase, beta-glucosidase, phosphate butyryltransferase P, UDP-N-acetylglucosamine acyltransferase, gluconate 5-dehydrogenase, and lactate permease (respectively *uxuB*, *galK*, *aguA*, *uidA*, K01188, *ptb*, *lpxA*, *idnO*, and *lctP*) were proportionally more abundant in efficient animals (lower FCR, **Supplementary Figure S1A**). The microbial gene lactoylglutathione lyase (*glo1*) is also associated with carbohydrate metabolism and identified for predicting FCR, but it had higher relative abundance in less efficient animals (higher FCR). Microbial genes *galK* and *xylE* (i.e., MFS transporter, SP family, xylose:H+ symporter) were both located in cluster 5, but this cluster was not significantly enriched for microbial genes associated to FCR. On the other hand, cluster 25 was enriched due to the presence of microbial genes *uxuB* and *lpxA*.

Microbial genes associated with amino acid metabolism and transport pathways were identified for the prediction of ADG and found to be relatively more abundant in animals with higher ADG (see **Supplementary Figure S1B**), e.g., aspartate-semialdehyde dehydrogenase and phenylacetate-CoA ligase (*asd* and *paak*, respectively). Some housekeeping genes were also identified for this set, including large subunit ribosomal protein L17 and L36, F-type H+-transporting ATPase subunit delta and FKBP-type peptidyl-prolyl cis-trans isomerase *slyD* (*rplQ* and *rpmJ*, *atpH*, and *slyD*). Genes *rplQ*, *atpH*, and *slyD* were relatively more abundant in animals with higher ADG, and *rpmJ* was relatively more abundant in animals with lower ADG. The microbial gene N-acetylmuramoyl-L-alanine amidase (*amiABC*) was identified for prediction of ADG, being negatively correlated with the trait.

All microbial genes simultaneously identified for predicting FCR and ADG showed a negative correlation to FCR and a positive correlation to ADG. These included housekeeping genes (*infA*, *hupB*, and *dph2*), a gene related to carbohydrate metabolism (*gcvH*), *murD*, which was associated with peptidoglycan metabolism and D-glutamine and D-glutamate metabolism, and *nusA*, associated with transcription regulation. Cluster 21 was enriched in ADG- and FCR&ADG-predicting microbial genes due to the presence of *atpH*, *rplQ* (ADG), and *infA* (FCR&ADG).

Five microbial genes identified for the prediction of RFI were associated with environmental sensing, bacterial chemotaxis, and motility: sensor kinase *cheA*, response regulator *cheY*, methyl accepting chemotaxis protein, flagellar motor switch protein *fliN/fliY*, and flagellar hook protein *flgE* (*cheA*, *cheY*, *mcp*, *fliN*, and *flgE*, respectively) were found to be relatively more abundant in more efficient animals, i.e., lower RFI. Other microbial genes associated with RFI are involved in the biosynthesis of cofactors and vitamins, particularly vitamin B12 production, for example, cobalt transport protein, threonine-phosphate decarboxylase, and precorrin-6Y C5,15-methyltransferase (decarboxylating), which correspond respectively to *cbiN*, *cobD*, and *cobL* (**Supplementary Figure S1C**). Finally, three genes that encode proteins related to carbohydrate transport and metabolism were relatively more abundant in more efficient animals (i.e., lower RFI): the simple sugar transport system permease protein, oxaloacetate decarboxylase, alpha subunit, and aldehyde:ferredoxin oxidoreductase (respectively ABC.SS.P, *oadA*, and *aor*). Cluster 4 was significantly enriched in microbial genes associated with RFI due to the presence of microbial genes *cobD*, *cobL*, *mcp*, and *oadA*, and serine-type D-Ala-D-Ala carboxypeptidase (penicillin-binding protein 5/6), inner membrane protein, and Cd2+/Zn2+-exporting ATPase (respectively, *dacC*, *ybrG*, and *zntA*).

The set of microbial genes identified for prediction of DFI included four microbial genes, proportionally more abundant in animals with higher DFI, which encoded proteins associated with environmental sensing, i.e., nitrogen regulatory protein P-II 1, outer membrane channel protein *TolC*, and preprotein translocase subunit *YajC* (*glnB*, *tolC*, and *yajC*, respectively). Nitrate reductase 1, alpha subunit (*narG*) was related to denitrification, releasing nitrite, and it was found to be relatively more abundant in animals with lower DFI (**Supplementary Figure S1D**). DNA-directed RNA polymerase subunit beta (*rpoB*, proportional higher abundance in animals with lower DFI), ribosomal large subunit pseudouridine synthase B, exodeoxyribonuclease VII small subunit, ribonuclease III, N utilization substance protein B, and integration host factor subunit alpha (respectively *rluB*, *xseB*, *rnc*, *nusB*, and *ihfA*, proportionally more abundant in animals with higher DFI) are housekeeping genes identified in this work for the prediction of DFI. Cluster 2 was significantly enriched with microbial genes associated with DFI due to the presence of *glnB*, *infA*, *mrdA*, *nusB*, *rdgB*, *rluB*, *tolC*, and *xseB*.

RFI- and DFI-predicting genes include glucose-1-phosphate cytidylyltransferase, CDP-glucose 4,6-dehydratase (respectively *rfbF* and *rfbG*, related to amino sugar and nucleotide sugar metabolism), and energy-converting hydrogenase B subunit D (*ehbD*, housekeeping). These three genes were proportionally more abundant in less efficient animals (higher RFI associated with increased DFI).

## Discussion

### Rumen Microbial Gene Abundances Associated With Efficiency Traits

**Figure 6:**
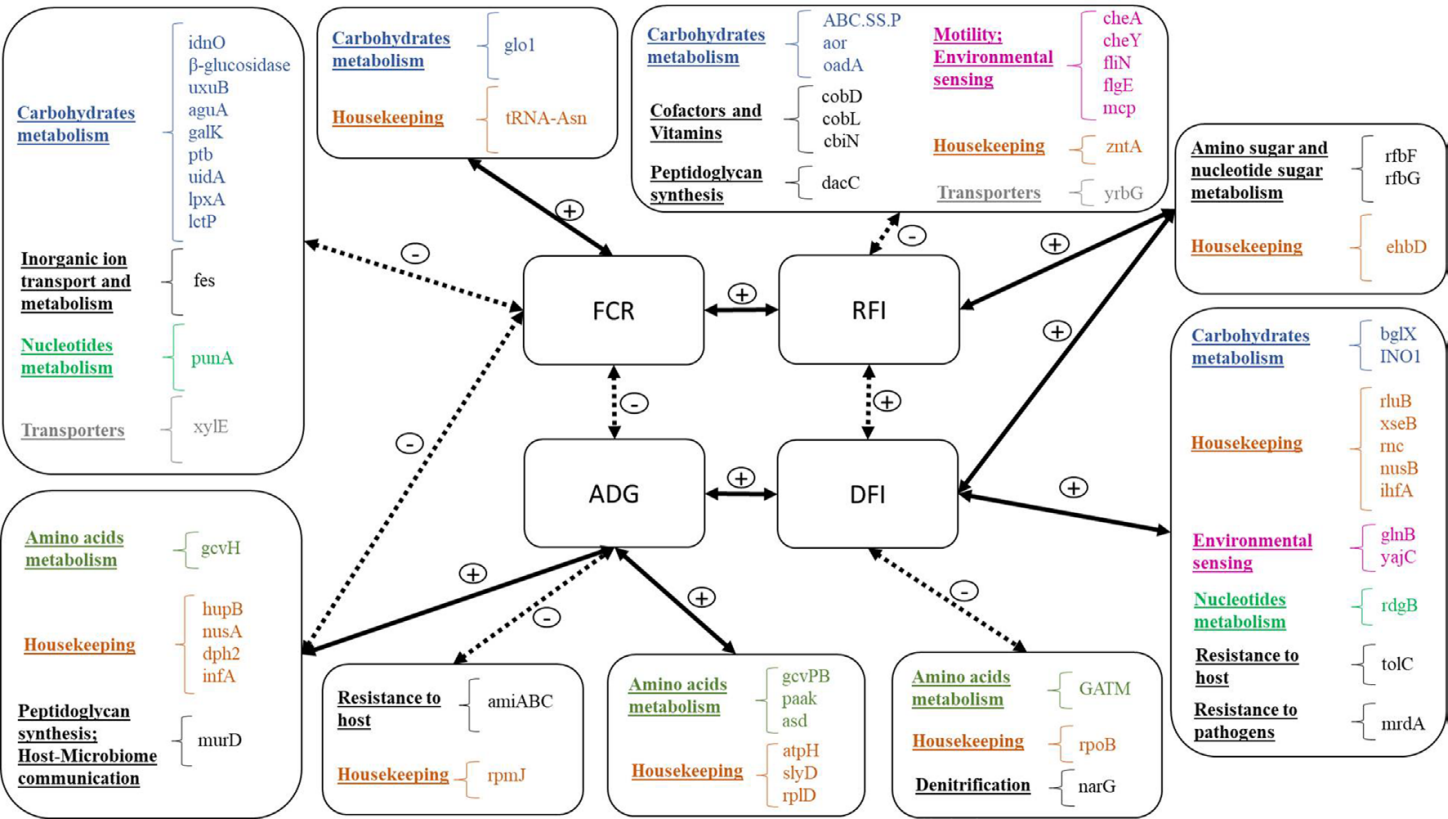
Summary of microbial genes identified for the prediction of each trait: Traits are located in the four central boxes: FCR, feed conversion ratio; ADG, average daily gain; RFI, residual feed intake; DFI, daily feed intake. Solid lines represent positive correlations, and dotted lines represent negative correlations. Microbial genes are listed in the outside boxes, organized by general function, and each general function is represented by a different color.

Our research indicates that there is a substantial link between rumen microbial gene abundances and appetite (measured as feed intake), growth rate, and feed conversion efficiency (**Figure 6**). The relative abundances of 20 and 17 microbial genes accounted for substantial variation (>60%) in FCR and RFI, respectively. The discriminant analyses of high- and low-performing animals indicated that accurate classification (>85% correct assignment of FCR and RFI categories) could be achieved using the microbial genes identified in the PLS for the prediction of the traits. Roehe et al. (2016) also found an association of microbial gene abundances with FCR, but their results were based on a smaller number of animals selected for their extreme values in methane emissions. In the present study, animals were selected based on their extreme FCR values, yielding a statistically more powerful estimate of this trait. Whereas FCR is calculated as a ratio between DFI and ADG and is therefore highly affected by growth rate and body composition, RFI is independent of these traits (Berry and Crowley, 2013). The low phenotypic correlation (*r* = 0.32) between FCR and RFI suggests that these traits capture substantially distinct characteristics.

For ADG and DFI, the relative abundances of 14 and 18 microbial genes, respectively, also explained substantial variation (>65%), and the discriminant analyses of high- and low-performing animals resulted in high prediction accuracies of 79 and 86%, respectively. These component traits were moderately correlated, agreeing with the report by Berry and Crowley (2013) of a large independent variation of feed intake and weight gain.

The animals’ appetite, feeding behaviour, and gastrointestinal motility (among other traits) are thought to be regulated by several mechanisms, including a communication between the rumen microbiome and the brain, through the gut–liver–brain axis (vagus nerve). This communication has been proposed to be mediated by multiple mechanisms, such as insulin/glucagon homeostasis, oxidation of acetyl coenzyme A, and release of VFA by the rumen microbiota (like propionate, associated with hypophagic behavior in ruminants, or butyrate and acetate, associated with motility of the gastrointestinal tract in monogastric animals; Sakata and Tamate, 1979; Cherbut, 2003; Oba and Allen, 2003; Arora et al., 2011; Maldini and Allen, 2018). Given the predictability of performance traits using relative abundances of rumen microbial genes observed in the present research (particularly that of DFI) and the high impact of the rumen microbiome on feed intake regulation (as discussed in the literature), we hypothesize that rumen microbial genes are closely involved in the metabolic pathways that regulate feed intake.

### Differential Microbial Gene Sets Predicting Distinct Trait Complexes

The coabundance microbial gene network (**Figure 5**) identified two separate trait complexes. While microbial genes identified for the prediction of FCR were grouped with ADG-predicting genes, microbial genes identified for the prediction of RFI were grouped with DFI-predicting genes, as revealed by differential enrichment in separate clusters (**Supplementary Figure S2**). For example, beta-glucosidase is encoded by microbial genes *bglX* and K01188, which were associated to different traits (DFI and FCR, respectively). This type of differential clustering was previously observed for microbial genes associated with methane emissions and FCR by Roehe et al. (2016). The trait complexes associated with feed conversion efficiency were further evidenced when analyzing the overlapping genes identified for the prediction of each trait (**Figure 4** and shaded rows in **Tables 2**–**5**), i.e., six microbial genes were identified for the prediction of both FCR and ADG and three genes for the prediction of both RFI and DFI. In agreement, strong correlations were observed for each pair of traits, as shown previously in the literature with the literature (Arthur and Herd, 2008; Herd et al., 2014). These results suggest that different microbial genes can be used to predict each trait. Furthermore, microbial genes overlapping for the prediction of more than one trait might be useful for the interpretation of biological processes explaining the correlation between phenotypes.

### Metabolic Pathways of Microbial Genes Associated With Efficiency Traits

Our results indicate that most proteins encoded by microbial genes identified for the prediction of FCR were generally involved in carbohydrates metabolism and transport. For example, *aguA* and K01188 are involved in biomass conversion, through the degradation of hemicelluloses and lignocelluloses and lactate biosynthesis (Cairns and Esen, 2010; Lee et al., 2012; Michlmayr and Kneifel, 2014; Li, 2015). Microbial genes *xylE*, *aguA*, and *uidA* are involved in xylan degradation, the main component of hemicellulose (Lee et al., 2012; Fliegerova et al., 2015). Xylose needs to be taken up by a transporter (putatively associated with *xylE*) before it is metabolized, and it has been recognized as a rate-controlling step in bacterial metabolism (Chaillou and Pouwels, 1999). Furthermore, microbial genes such as *uidA* [previously identified by Roehe et al. (2016)], directly involved in carbohydrate metabolism pathways like pentose and glucuronate interconversions and galactose metabolism, are coupled with NAD or NADP oxidoreduction, important for regulating the flux of carbon and energy sources in microorganisms (Spaans et al., 2015). In addition, *punA* (i.e., purine-nucleoside phosphorylase) is involved in the metabolism of nucleotides, nicotinate and nicotinamide (vitamin B3), which also contain NAD and NADP, and is therefore important in carbohydrate, protein, and lipid metabolism reactions. Positive effects of vitamin B3 have been previously observed in healthy rumen microbiomes in beef and dairy cattle (Aschemann et al., 2012; Luo et al., 2017). Microbial genes *uidA* and *punA* were more abundant in efficient animals.

Proteins encoded by *lctP*, K01188, and *ptb*, involved in lactate transport and cellulose and butyrate metabolism, respectively, could be involved in host–microbiome crosstalk mechanisms in cattle due to their participation in metabolic pathways that involve the release of H^+^, such as lactate metabolism, potentially reducing microbial fiber-degrading activity and consequently slowing digestion and rumen emptying rate, causing a decrease in appetite (Moran, 2005b). Furthermore, beta-glucosidase is widely present in lactic acid bacteria and is thought to interact with the human host (Michlmayr and Kneifel, 2014). Butyrate has been shown in rats to directly activate the intestinal gluconeogenesis genes in enterocytes *via* an increase in cationic antimicrobial peptides (cAMP, De Vadder et al., 2014). In contrast, *glo1* (more abundant in FCR-H) is involved in methylglyoxal degradation, which is a highly toxic substance that decreases bacterial cell viability, and is produced by bacteria when there is carbohydrate excess and nitrogen limitation (Russell, 1993). Therefore, *glo1* is a strong candidate biomarker of rumen microbiome difference in less efficient animals (i.e., FCR-H).

The microbial gene with highest impact in prediction of ADG was *amiABC*, which is mainly involved in the peptidoglycan turnover through cleavage of glyosidic bonds and release of amino acids and cAMP resistance (Uehara and Park, 2008; Uehara et al., 2010). Some bacteria (mostly pathogenic) have evolved mechanisms of resistance, such as decreased affinity to cAMPs (Anaya-López et al., 2013), and the higher abundance of *amiABC* in animals with lower ADG may be indicative of higher abundance of pathogens, which can cause inflammatory response in the rumen potentially reducing nutrient use and absorption (Reynolds et al., 2017). Brown et al. (2003) demonstrated that acetate and propionate are agonists of the human receptors GPR43 and GPR41, and Hong et al. (2005) proposed that acetate and propionate induce lipid accumulation and inhibition of lipolysis through the GPR43 receptor in mice. These genes are also part of the bovine genome, where they mediate an inhibitory effect of acetate, propionate, and butyrate on cAMP signaling (Wang et al., 2009). This could indicate that, in less efficient animals (lower ADG), the lower amount of acetate, propionate, and butyrate may lead to decreased inhibition of lipolysis by the host, which potentially results in lower ADG. Alternatively, the lower amount of VFAs in these animals may lead to decreased inhibition of cAMP signaling and increased release of cAMPs by the host to the rumen. The cAMPs act primarily on organisms without effective resistance mechanisms, consequently increasing the relative abundance of cAMP-resisting organisms and of the microbial genes encoding for the resistance. Two other microbial genes identified in the present research are part of the cAMP resistance pathway—*lpxA* and *tolC* (associated with FCR and DFI, respectively). Although all three genes (*amiABC*, *lpxA*, and *tolC*) are part of the same pathway, they present opposite tendencies—while *lpxA* and *tolC* are proportionally highly abundant in animals with higher ADG and lower FCR, *amiABC* is relatively highly abundant in animals with lower ADG and higher FCR. The gene *lpxA* is related to lipid A integration in the cell wall, as a preventive measure against the hosts’ immune system, and *tolC* is involved in the efflux of antibiotics (Raetz et al., 2007; Zgurskaya et al., 2011). This could be indicative of the different cAMP resistance mechanisms evolved by bacterial organisms, which include modification of the cell external surface, efflux pumps, and biosynthesis and crosslinking of cell envelope components (Nizet, 2006).

The set of microbial genes associated with ADG included mostly housekeeping genes and genes related to amino acid metabolism and transport. Artegoitia et al. (2017) found a link between ruminal aromatic amino acids synthesis such as phenylalanine and high ADG in beef steers. For example, *paak* [previously mentioned by Kamke et al. (2016) related to sheep with high production of methane] and *asd* encode proteins that respectively catalyze phenylalanine and phenylacetate (related to aspartate degradation and biosynthesis of amino acids including threonine), with release of H^+^. In the current research, both of these genes were positively correlated to ADG, which is supported by the positive correlations between ADG and dry matter intake (DMI), between DMI and methane emissions, and between methane emissions and body weight measurements (weaning weight, yearling weight, and final weight), previously observed in cattle (Koots et al., 1994; Arthur et al., 2001; Herd et al., 2014).

Some housekeeping genes were simultaneously identified for the prediction of FCR and ADG, such as protein translation from diphthamide (*dph2*) or peptidoglycan biosynthesis (*murD*), both more abundant in efficient animals (higher ADG and lower FCR). The importance of diphthamide biosynthesis in archaea is not yet fully known (Narrowe et al., 2018). Microbial gene *murD* is related to the glutamate–glutamine cycle, an important appetite regulator in humans (Delgado, 2013), but in the present research, it was not associated to DFI.

Proteins encoded by microbial genes associated with RFI are mostly related to chemotaxis (*cheA* and *cheY*), detoxification (Cd2+/Zn2+-exporting ATPase, *zntA*), and vitamin B12 production (*cbiN*, *cobD*, and *cobL*). The negative correlation of microbial genes involved in chemotaxis and motility with RFI may suggest an increased microbial metabolism in efficient animals, derived from their ability to sense chemical gradients in their surrounding environment and to react accordingly, i.e., moving closer to nutrients (Rajagopala et al., 2007). Microbial gene *zntA* was also more abundant in efficient animals and plays a role in the homeostasis of transition metals (Cd^2+^, Zn^2+^), participating in functional pathways ranging from cellular respiration to gene expression (Fraústro da Silva and Williams, 2001). Finally, higher relative abundance of microbial genes involved in vitamin B12 production (*cbiN*, *cobD*, and *cobL*) was observed in more efficient animals. This essential cofactor needs to be taken up directly from the diet or to be made available for animal absorption by the rumen microbial organisms because it is not produced by eukaryotes (Warren et al., 2002). Furthermore, vitamin B12 has been previously associated with increased cobalt content on high-fiber diets and increased VFA, such as acetate (Beaudet et al., 2017), which may affect the animals’ appetite (Frost et al., 2014), in line with our observation of higher relative abundance of these genes in more efficient animals, i.e., animals with lower feed intake than expected.

The four most important microbial genes identified for the prediction of DFI included the three microbial genes also identified for prediction of RFI (*rfbG*, *rfbF*, and *ehbD*) and *narG*. Microbial genes *rfbG* and *rfbF* (VIP > 1.4) are part of the *rfc* region (Morona et al., 1994) and are related to nucleotide sugar metabolism, which is necessary for the production of microbial lipopolysaccharide (LPS). LPS is a major virulence factor of Gram-negative bacteria, particularly due to the O-antigen, paramount for host colonization and niche adaptation by bacterial organisms, due to its part in the protection from host immune response (Reeves, 1995; Samuel and Reeves, 2003; Geue et al., 2017). Both genes *rfbG* and *rfbF* showed a positive correlation to RFI and DFI, supporting our hypothesis that the use of energy to stimulate the innate immune system against pathogens increases DFI and reduces feed conversion efficiency as determined by RFI (Neal et al., 1991; Jing et al., 2014; Vigors et al., 2016). Other microbial genes positively correlated to DFI were found to be involved in resistance mechanisms, such as the penicillin-binding protein 2-encoding gene (*mrdA*), which belongs to the peptidoglycan and beta-lactam resistance metabolic pathways. These proteins are transpeptidases or carbopeptidases involved in peptidoglycan metabolism and have an important role against beta-lactam resistance (Zapun et al., 2008). The microbial gene myo-inositol-1-phosphate synthase (*INO1*) is related to antibiotic biosynthesis, including streptomycin. Microbial gene *ehbD* is a subunit of the energy-converting hydrogenase B, found in methanogens such as *Methanococcus maripaludis*. This microbial gene is important due to its role in autotrophic CO_2_ assimilation (Porat et al., 2006), having implications for microbial growth. Furthermore, *narG*, part of the *narGHIJ* operon, essential for some microorganisms to gather energy under anaerobic conditions by the reduction in nitrate to nitrite in a denitrification process (Blasco et al., 1990; Latham et al., 2016), was proportionally more abundant in animals with low DFI.

The microbial gene *nusB* (associated with DFI) is part of a set of *nus* genes, which also includes *nusA* (identified for prediction of FCR and ADG). Genes in the *nus* complex are involved in transcription termination and antitermination processes, such as Rho-dependent transcriptional termination (Torres et al., 2004), which is the regulatory mechanism involved in the efficient transcription of the tryptophan operon (Farnham et al., 1982; Kuroki et al., 1982; Prasch et al., 2009). The *nus*-complex microbial genes were found to be relatively more abundant in efficient animals. This association may be due to the influence of the *nus* genes, which extends from the ribosomal operons to the tryptophan operon and constitutes a good example of how termination and antitermination processes can control gene expression, occurring during RNA transcription, and potentially positively impacting bacterial growth and rumen fermentation processes.

Although microbial genes *amiABC*, *tolC*, *glo1*, *rfbF*, *rfbG*, *and lpxA* were identified in the present research for the prediction of different traits, all are associated with bacterial defense mechanisms either from other bacteria or from the host. The majority of these genes had higher abundance in less efficient animals. This suggests that the presence of either bacterial pathogens in the rumen or antibiotics produced as host immune responses might represent a significant energy sink, impairing feed conversion efficiency.

Further improvement of prediction of feed conversion traits using metagenomic information may be achieved through the integration of protein, enzyme, and pathway data from the Hungate collection (Seshadri et al., 2018) and the large rumen metagenomic reference dataset (Stewart et al., 2018).

## Conclusions

The results presented here suggest that relative abundances of rumen microbial genes may be highly informative predictors of feed conversion efficiency, growth rate, and feed intake, which are labor intensive, time consuming, and expensive traits to record. Most microbial genes identified for the prediction of traits in this research were trait specific. Microbial genes related to cellulose and hemicellulose degradation, vitamin B12 synthesis, and amino acids metabolism were associated to enhanced feed conversion efficiency (FCR or RFI), while those involved in nucleotide sugars metabolism, pathogen LPS synthesis, cAMP resistance, and degradation of toxic compounds were associated with inefficient feed conversion. Furthermore, we identified specific microbial genes encoding proteins related to the crosstalk between the microbiome and the host cells, such as *murD* and *amiABC*, and associated to gene expression regulatory mechanisms, such as *nusA* and *nusB*. Thus, our results provide a deeper understanding of the potential influence of the rumen microbiome on the feed conversion efficiency of its host, highlighting specific enzymes involved in metabolic pathways that reflect the complex functional networks impacting the conversion of feed into animal products such as meat.

## Author Contributions

RR and MW conceived and designed the overall study, and JL, MA, TF, and RR conceived, designed, and executed the bioinformatics analysis. RS and MW carried out the bioinformatics to obtain the rumen microbial gene abundances. C-AD, TS, RD, and AW provided essential insight into feed conversion efficiency, rumen metabolism, nutrition, and microbiology. JL, MA, and RR wrote the initial draft, and subsequently, all authors contributed intellectually to the interpretation and presentation of the results.

## Funding

The project was supported by grants from the Biotechnology and Biological Sciences Research Council (BBSRC BB/N01720X/1 and BB/N016742/1) and by the Scottish Government (RESAS Division) as part of the 2016–2021 commission. The research is based on data from experiments funded by the Scottish Government as part of the 2011–2016 commission, Agriculture and Horticulture Development Board (AHDB) Beef & Lamb, Quality Meat Scotland (QMS), and Department for Environment Food & Rural Affairs (Defra).

## Conflict of Interest Statement

The authors declare that the research was conducted in the absence of any commercial or financial relationships that could be construed as a potential conflict of interest.
