# Supplementary material for: Identification of Rumen Microbial Genes Involved in Pathways Linked to Appetite, Growth, and Feed Conversion Efficiency in Cattle
Source: Front Genet. 2019 Aug 8;10:701. doi: 10.3389/fgene.2019.00701 (PMC6694183; doi:10.3389/fgene.2019.00701)
Supplement: Supplementary file 1 [file Table_1.docx]

Supplementary Material

# Supplementary Figures

A)


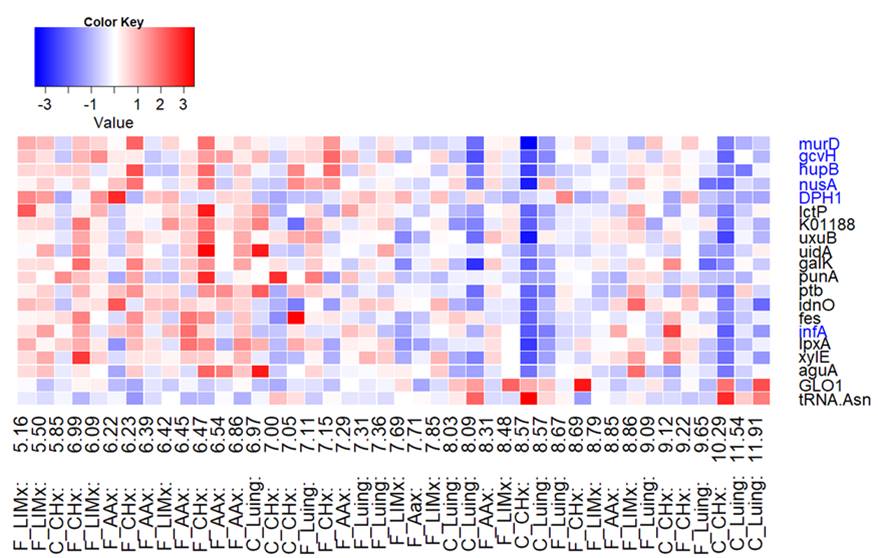


B)
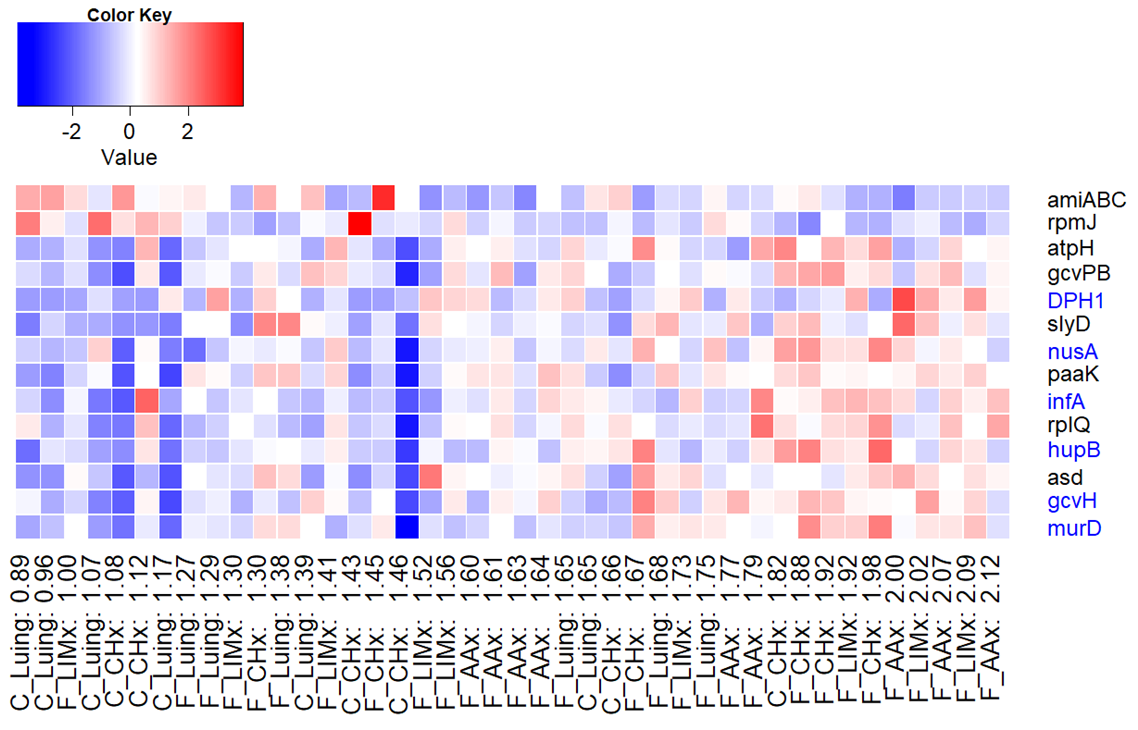


C)
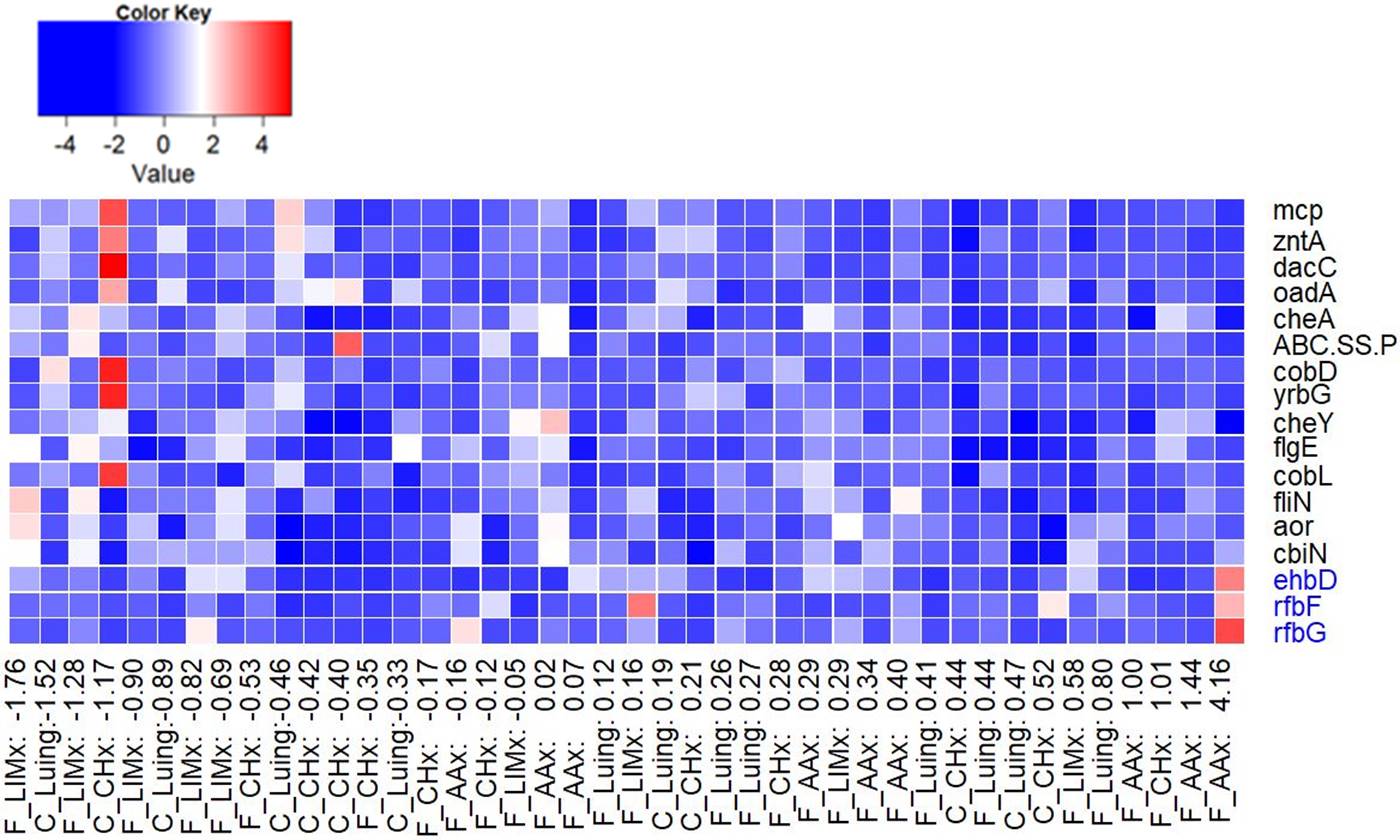


D)
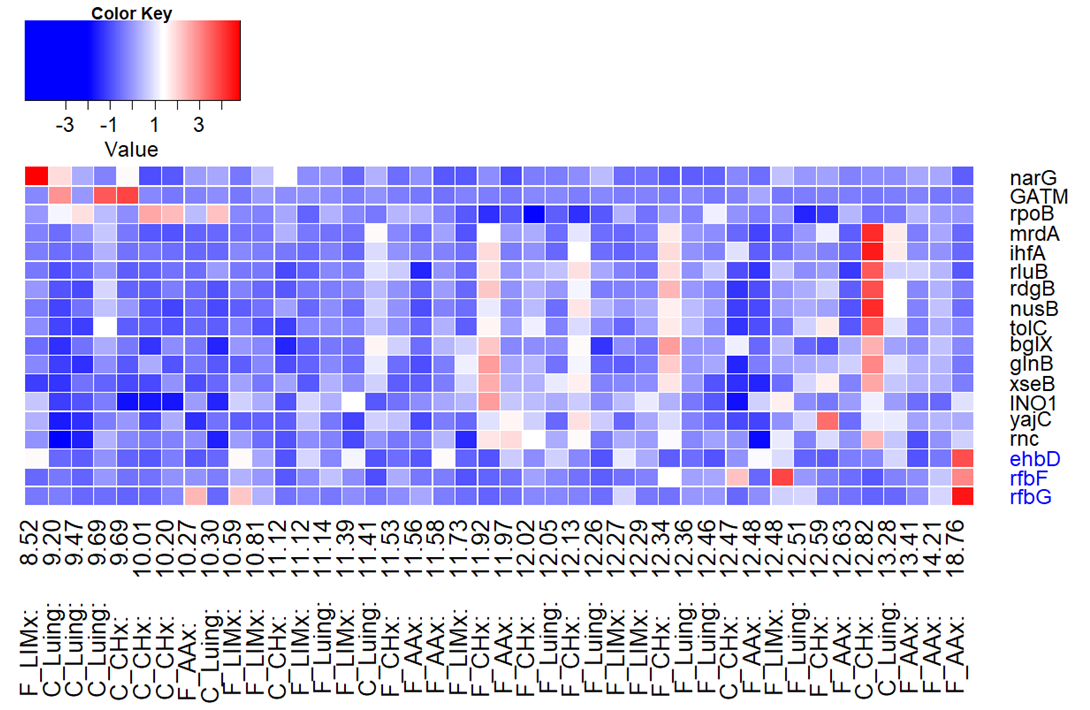


**Supplementary Figure S1.** Heatmaps of relative abundances of microbial genes identified for the prediction of A) feed conversion ratio, B) average daily gain, C) residual feed intake and D) daily feed intake. These microbial genes were identified through linear models and partial least squares analyses for the prediction of each trait. The relative abundance of the microbial genes (blue = low to red = high) change according to the animal: the labels on the horizontal axis represent each animal and contain information indicating the breed type (AAX – crossbred Aberdeen Angus, CHx – crossbred Charolais, LIMx - crossbred Limousin, Luing – purebred Luing), diet fed to the animal (F – forage, C – concentrate) and the approximate value of the trait. Microbial genes whose names are in blue were simultaneously identified for the pairs of traits FCR and ADG or DFI and RFI.


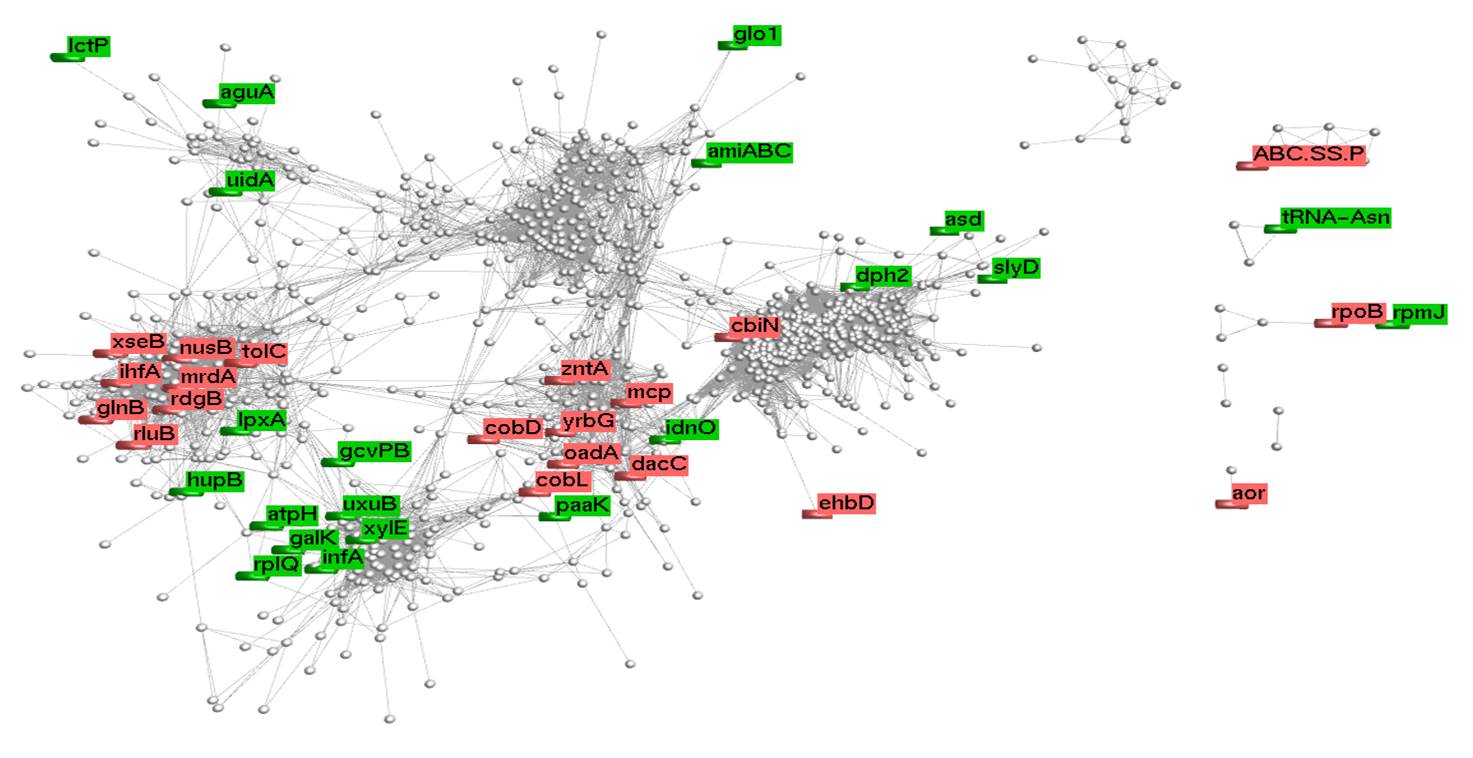


**Supplementary Figure S2.** Co-abundance network of microbial genes, evidencing the separation between microbial genes identified for the prediction of feed conversion ratio and/or average daily gain (represented in green), and residual feed intake and/or daily feed intake (represented in red). Each node represents a microbial gene and each edge represents a correlation higher than 0.80 between them.
